# Learning from Peers: A Qualitative Study to Inform the Development of a Community Tailored Peer Support Intervention to Support Healthy Infant Growth

**DOI:** 10.3390/nu17243941

**Published:** 2025-12-17

**Authors:** Colin J. Orr, Alexander Acosta, Luis Acosta, Aunchalee E. L. Palmquist, Carrigan Price, Jennifer Guterriez-Wu, Adriana R. Gaona, Edwin B. Fisher

**Affiliations:** 1 Department of Pediatrics, Vanderbilt University Medical Center, Nashville, TN 37232, USA; 2 Department of Pediatrics, University of North Carolina at Chapel Hill, Chapel Hill, NC 27599, USA; 3 Department of Pediatrics, The Johns Hopkins University School of Medicine, Baltimore, MD 21205, USA; 4 Duke Global Health Institute, Duke University School of Medicine, Durham, NC 27710, USA; 5 Peers for Progress, Department of Health Behavior, Gillings School of Global Public Health, University of North Carolina at Chapel Hill, Chapel Hill, NC 27599, USA

**Keywords:** food insecurity, pediatric obesity, peer parent coach, infant food insecurity, infant growth

## Abstract

Background: Obesity is a chronic disease that has negative health consequences for children. Peer support models have been used to manage chronic diseases like diabetes; however, little is known about how a peer support intervention might promote healthy infant growth to prevent pediatric obesity. The aim of this project was to explore parental perspectives on how a peer support intervention might be developed to support healthy infant weight gain and nutrition. Methods: Data were collected from November 2022 to October 2023 at a single pediatric primary care clinic. Semi-structured interviews explored parents’ perspectives of how a peer parent coach could promote healthy infant nutrition and growth. Interviews focused on (1) common infant feeding and nutrition questions, (2) the role and importance of peer support during the newborn period, and (3) strategies for addressing and facilitating connections to food-related resources and addressing food insecurity. Results: A total of 18 interviews were conducted. Average parental age was 32.1 years (range 20–46 years). Thirty-three percent of the participants identified as Black, 28% identified as White, 11% identified as Asian, and the remaining identified as Other or preferred not to report. Half of the sample reported a household income of <$20,000, 67% reported having public insurance, and 11% reported household food insecurity. Themes that emerged included: peer parent coaches can (1) provide emotional support to families with young infants, (2) education focused on infant nutrition, and (3) facilitate connections with nutrition resources. Participants also noted the importance of understanding a family’s unique culture when counseling on infant growth and nutrition. Conclusions: Multiple themes were identified about how a peer support intervention could support healthy infant nutrition and growth. Future work should test the feasibility and acceptability of a peer support intervention to promote healthy infant weight gain.

## 1. Introduction

The health of children in the United States has deteriorated over the last 2 decades [1]. One key indicator of this trend is the rising prevalence of pediatric obesity, which, according to data from 2023, now affects approximately 1 out of 5 children in the U.S. [1,2]. A higher prevalence of pediatric obesity is observed among Black children, Hispanic children, children from rural communities, and households with fewer socioeconomic resources [3].

Pediatric obesity is associated with numerous detrimental health conditions in childhood, including hypertension, depression, metabolic associated liver disease, glucose dysregulation, obstructive sleep apnea, musculoskeletal disease, and lipid disorders [4]. In addition to contributing to poor health in childhood, pediatric obesity can lead to long-term consequences that extend into adulthood. Nearly, 50% of children and 80% of adolescents with obesity are expected to meet criteria for obesity in adulthood [5]. Preventing obesity in childhood has the potential to improve pediatric health, with benefits that continue into adulthood, where obesity is associated with excess mortality [6].

The newborn period and early infancy represent a critical developmental window to promote the health of children [7]. The factors that contribute to a child’s risk of developing obesity can begin during infancy and continue across the life course [8]. Contributors to infant growth and nutritional outcomes, include parental feeding behaviors [9], parental mental health [10,11], and household food security status [12,13,14,15,16]. Parental feeding behaviors that may increase a child’s risk of obesity can present as early as 2 months of age and the velocity of infant weight gain during the first 9 months of life is a key contributor to the development of obesity in childhood [17,18]. Implementing interventions that target the drivers of infant growth and nutrition is a strategy to improve the health of children by preventing obesity.

Peer support-based interventions represent a promising approach. Peer parent interventions bring together people with shared experiences to help manage health conditions, facilitate connection with community resources, and provide emotional support [19]. In the U.S., such interventions have demonstrated success in improving management of chronic conditions, such as pediatric asthma, increasing Medicaid enrollment, and supporting breastfeeding practices among mothers with fewer socioeconomic resources [20,21,22,23,24].

The potential for peer support interventions to improve the nutrition and growth of infants remains underexplored, particularly among households with lower socioeconomic status, Black and Hispanic communities, or communities experiencing FI. To achieve this, it is important to understand how such an intervention can best support families and healthy infant weight gain. A community tailored peer parent support intervention, in which peer parent coaches (PPCs) work alongside families of newborns to support healthy infant nutrition and weight gain, may offer a promising strategy to promote healthy infant growth for all families, especially those who face barriers to health.

Toward that end, the objective of this study was to explore the perspective of families with young infants, in order to inform the development of a novel peer parent coach intervention.

## 2. Methods

### 2.1. Study Setting

The study was conducted at a single pediatric primary care clinic located in the southeastern United States between November 2022 and October 2023. The clinic is affiliated with an academic medical center and serves a geographically, economically, racially, and ethnically diverse patient population. Approximately 70% of children served by the clinic are publicly insured, 30% of families identify as Hispanic, 30% identify as Black, 30% identify as White, and 10% identify as another race or ethnicity The clinic serves families from five counties that represent urban and rural communities. Since 2016, the clinic has routinely screened for FI using a validated 2-item screening tool [25] available in English and Spanish. Families who screen positive for FI are offered resources including: (1) access to an on-site emergency food pantry, (2) a list of community food resources, and (3) referral to WIC when appropriate.

### 2.2. Study Participants and Recruitment

The study sample was a convenience sample of children and their parents who presented to the pediatric primary care clinic for a routine well-child visit. Parental inclusion criteria included: (1) parent of a child 0–6 months of age, (2) age ≥ 18 years, and (3) fluency in English or Spanish. Child inclusion criteria included: (1) age between 0–6 months, (2) born at ≥36 weeks gestation, and (3) the absence of known medical conditions that could affect growth (e.g., renal disease, genetic conditions, clinically significant cardiac disease, etc.). The study focused on children ≤ 6 months of age due to the evidence noted above of the importance of this early developmental period [7,17,18]. Potentially eligible parent-infant dyads were approached by one of two bilingual research assistants (LA and AA) during the course of a standard well child check appointment. Participants were approached for potential participation in the study if the parent-infant dyad was presenting to clinic for a 2-, 4-, or 6-month well child check appointment. Parent-infant dyads were approached in the privacy of a clinical exam room. Eligible parents who expressed interest were consented, provided verbal consent, and were enrolled in the study. Interviews were scheduled at the time of enrollment in the study or within a week. The study was approved and deemed exempt by the institutional review board at Vanderbilt University IRB (#2413872, 14 January 2025) and Chapel Hill IRB (#’s 22-2319, 15 May 2023, and 22-1410, 24 October 2022).

### 2.3. Study Design

Data for the study were collected via individual semi-structured interviews with participants. Interviews were conducted by members of the study research team (AA, LA, CP), all of whom were trained in qualitative methods (MG). Specific questions included in the interview guide were informed by a review of the literature and expert opinion (Table 1).

To ensure quality assurance, the principal investigator (Orr) observed a subset of interviews and provided individualized feedback to interviewers. The interviews were designed to last 45–60 min and conducted virtually, either by a HIPAA compliant Zoom or phone call. Interviews were conducted in English or Spanish, according to the caregiver’s language preference, and facilitated by interviewers who were fluent in the respective language. Concepts explored throughout the semi-structured interviews included: (1) common infant feeding and nutrition questions that a peer parent coach intervention could address (e.g., “How might a PPC provide support for bottle- or breastfeeding?”), (2) the role and importance of peer support during the newborn period (e.g., “How might a peer support coach provide emotional support to parents?”), and (3) strategies for addressing FI and facilitating connections to food-related resources (e.g., “What resources do you think a peer parent coach would need to support families who experience FI or need help enrolling in WIC or SNAP?”). Interviewers asked probing questions based on participants’ responses. To ensure interviews ran smoothly and were comfortable for participants, a pilot interview was conducted in English. Adaptations were made to the interview guide based on observations and findings from the pilot interview; however, these findings were not included in the final analytical sample.

### 2.4. Demographics

Caregivers were asked to report their age, gender (male, female, other), relationship to child (mother, father, grandmother, grandfather, other), highest level of education, primary language spoken at home (English or Spanish), number of children living in the home, annual household income, and insurance status (public/Medicaid/Managed Care, private, uninsured, other). Caregivers were asked to self-report their race and ethnicity. Available responses for race included: Black or African American, White, Asian, Pacific Islander, American Indian/Alaskan Native, and a free-text option. Ethnicity was categorized as Hispanic, non-Hispanic, or free-text option. FI was determined based on the screener by Hager et al. [25], consisting of two questions: “We worried whether our food would run out before we got money to buy more” and “The food we bought just didn’t last, and we didn’t have money to get more.” [25]. Response options included: often true, sometimes true, and never true. Participants reporting often true or sometimes true to at least one of the questions were classified as “food insecure”. Participation in the Special Nutrition Program for Women and Children (WIC) was assessed by the questions “Are you enrolled in WIC (for your child or yourself)”. Response categories included “yes to both”, “yes for child only”, “yes for me (caregiver only)”, and “no”.

### 2.5. Analysis

Descriptive statistics were used to summarize demographic characteristics. The objective of the study was to explore parent’s perspectives on how a PPC could support healthy infant weight gain and nutrition based on hypothesized factors that contribute to infant growth and nutrition. To achieve this goal, a combination of deductive and inductive analyses were used [26]. The Social Determinants of Health framework was used to inform the development of initial codes [27,28]. The Social Determinants of Health framework was chosen given the study’s aim of understanding the factors outside of the health care environment that influence infant growth and nutrition such as socioeconomic status, knowledge of infant of growth and nutrition, community characteristics, and access to food and nutrition [27]. After collection and transcription of data, transcripts were reviewed for the emergence of novel themes that were not included in the a priori codes [29]. New themes were added to the coding guide as needed. All interviews were recorded and transcribed verbatim [30]. All transcripts were coded independently by two coders. Coding discrepancies were discussed with the full research team until a final coding decision was made. For interviews conducted in Spanish, transcripts were transcribed and doubled-coded in Spanish by members of the research team who were native Spanish speakers (AA, LA, JGW, ARG). Themes and subthemes were identified through an iterative process, and representative quotes were selected. As needed, quotations were translated into English for reporting purposes. Data collection continued until thematic saturation was reached, defined as the point at which no new themes were identified [30]. Thematic saturation was determined after a review of 16 transcripts, at which point and no novel themes were identified. The research team elected to complete two additional interviews to ensure that thematic saturation had been reached. As a pilot study exploring and informing a peer parent coach intervention we were also interested in theoretical saturation where additional interviews did not identify new themes within the coding framework [28]. Reflexivity was evaluated during the study through individual reflection as well as peer debriefing sessions between members of the research team [31]. Topics discussed included cultural and language similarities or differences with participants, discomfort when discussing sensitive topics such as experience with food insecurity, and recognition of differences in lived experiences such as never having breastfed an infant. Multiple coders and a group process to understand convergence and divergence in coding and development of themes was used to mitigate bias in the analytical process. Data analysis was conducted via Dedoose Version 9.0. 107, cloud application for managing, analyzing, and presenting qualitative and mixed method research data (2023), Los Angeles, CA [32].

## 3. Results

### 3.1. Participant Demographics

A total of 30 parent–infant dyads were approached for potential participation in the study. Twenty parent–infant dyads met eligibility criteria and consented to participation in the study. A total of 18 interviews were conducted. Seventeen participants identified as a mother and one participant identified as a father (Table 2). The average age of the caregivers was 32.1 years (range 20–46), and 10 participants reported having a high school degree or less. Six participants identified as Black, five identified as White, two identified as Asian, and five did not respond or listed other. Six reported their ethnicity as Hispanic and four reported Spanish as their primary language. Two participants reported household FI, half reported an annual household income of <$20,000, 10 reported participation in WIC, and 12 reported having public insurance.

### 3.2. Interview Themes

Four themes emerged from the interviews:

- Theme A: Opportunity exists for PPCs to emotionally support families;
- Theme B: Educational support focused on infant nutrition is needed;
- Theme C: Facilitating connection with nutrition resources;
- Theme D: The importance of understanding a family’s culture and lived experience.

The following sections describe each of these themes in more detail, and quotations from interviews are presented in Table 3, Table 4 and Table 5 to elaborate upon them.

Theme A: Emotional Support

A majority of participants identified that a PPC could support families by providing emotional support (Table 3). This need was particularly salient during the transition period from the hospital to life at home with a newborn:

*“…but the moral support would be a huge advantage. Just not feeling too overwhelmed and alone in those first couple [of] weeks because you’re dismissed from the hospital and you’re like ‘Well what the heck is going on’”.*

(Participant 1)

Many participants felt that individuals with shared experiences, particularly those who had recently gone through the newborn phase of parenting, were well positioned to provide emotional support.

*“So I feel like I wanted someone there with me at all times who had gone through what I was going through”*

(Participant 2)

Respondents noted that while having knowledge is important, a PPC’s genuine interest and emotional presence may be even more valuable in supporting families:

*“But I think in terms of being helpful in an educational way, I’m not sure if it would’ve necessarily been like, “Can you instruct me on how to do something?” It’s more just like moral support and curiosity and empathizing with people.”*

(Participant 1)

Although 17 of the 18 participants identified as mothers, one mother highlighted the importance of emotional support for fathers, who may also be affected by the rapid changes associated with welcoming a newborn home:

*Emotional ... I mean not I would say me but I guess his dad maybe. It might be challenging on him. I mean it’s all new for both of us but he’s been having more difficulties more than me adjusting.*

(Participant 3)

Theme B: Educational Support Focused on Infant Nutrition

Several participants reported that a PPC could play a role in providing education on infant feeding and nutrition. A common area of interest among study participants was identifying hunger cues and understanding appropriate types and quantities of foods to feed their infant (Table 4). A few participants specifically noted opportunities to improve infant nutrition through interactions with a PPC:

*“I would say definitely giving guidance on nutritional things, on food. Like, on what’s more important, proteins and vegetables and stuff, a lot of people have the general idea, but not as much as it seems”*

(Participant 4)

In addition to education on infant nutrition, a small number of participants identified the importance of demonstrating the logistics and details of feeding such as formula preparation or breastfeeding:

*“So I really do mean—it’s not demonstrating* [breastfeeding] *‘cause they’re not demonstrating on themselves but it’s being able to lead you through physically the things that happen and things that can help in your case. And then of course since we had to do formula, there was also the logistical things of how do you prepare formula and these are not so hard but it was still helpful to have someone do that.”*

(Participant 5)

At least one participant emphasized the importance of tailoring guidance and education to align with the resources available to the family:

*“When you’re low-income too, it’s hard to be exposed or provided the better nutrition stuff. So, it would be good to guide lower-income families on what they can afford, and what’s important to provide to their kids.”*

(Participant 4)

An interest in the potential education that a PPC could provide was motivated by parents’ desire to learn about infant growth and nutrition:

*“most definitely if that parent wants to learn, and would be acceptable of learning different things or hearing different things, and trying different things. Not even different things—just sometimes you can have the same food with different ingredients, and you know less proportions, or different proportions, or different starches with this protein. A talk like that.”*

(Participant 6)

Theme C: Facilitating Connection with Nutrition Resources

For families who experience FI or facing barriers to optimal nutrition, federal nutrition programs such as the Special Supplemental Program for Women, Infants, and Children and the Supplemental Nutrition Assistance Program (SNAP) can play an important role in addressing FI and improving the health of children and their families.

Participants noted that although federal nutrition programs are intended to support families experiencing FI, or who have limited access to nutritious foods, awareness of these programs may be limited among eligible families. Participants identified PPCs as potentially valuable in facilitating resource connection by increasing awareness and understanding of these programs (Table 5). A theme that was shared among both English and Spanish speaking participants was apprehension about eligibility requirements and the benefits of participating in federal nutrition programs. Participants viewed PPCs as well positioned to address these concerns:

*“I was very apprehensive to even enroll in WIC and stuff, but it was a very easy process, and it helps out greatly. You don’t really think that it’s going to contribute that much, but it’s nice to know that you at least have milk and vegetables and the formula for your kid and stuff. I guess, don’t be afraid to reach out and get help is my biggest point”*

(Participant 4)

Participants also noted that access to accurate information about participation in federal nutrition programs is critical for reducing barriers to participation:

*“Pues más que nada informarlos bien, como, darles exactamente la información correcta. Porque hay veces que te meten miedo, como que: “Ay, si agarras esto, eh, estos programas, este, pues a futuro te traen consecuencias o problemas.” Hay gente que así-así le hace. Entonces más que nada informar a la gente con la verdad. Como, los procesos, qué se necesita, el tiempo que necesitas para los trámites y-y todo eso. Más que nada hablar con la verdad. Con la verdad y-y ser lo más, lo-lo más explícitos porque a veces, este, uno no entiende, como, toda la información o a veces te la hacen como muy revoltosa. Entonces entre menos palabras y más claros, mejor.”*

(Participant 7)

*English Translation: “Well, more than anything, to inform them well, like, give them exactly the correct information. Because sometimes they scare you, like: ‘Oh, if you receive this, uh, these programs, uh, well in the future it’ll bring you consequences or problems.’ There are people who do do that. So more than anything,* *inform people with the truth. Like, the process, what’s needed, the time you need for the paperwork and—and all that. More than anything, speak the truth. With the truth and—and be as—as explicit as possible because sometimes, uh, you don’t understand, like, all the information or sometimes they make it all really confusing. So the fewer words and the clearer, the better.”*

Theme D: The Importance of Understanding a Family’s Culture and Lived Experience

In addition to the three themes described above, an important theme identified by participants was the importance of understanding families’ unique cultures, lived experiences, and support systems. Understanding these factors can help ensure the support provided to families is responsive to their specific needs.

*“So, I think, a peer parent should be able to get to understand, you know, different models from different backgrounds and all that. So, when he or she goes there, she’ll be able to relate, because you know, from culture to culture, it’s different.”*

(Participant 16-participant is from Africa)

Participants also noted that different cultures can have different expectations around infant feeding.

*“So in the Mexican culture, they tell you that it’s better to breastfeed your child than to give them bottle. So I would feel bad with [NAME] because she didn’t want to breastfeed so I had to give her a bottle. And they would always ask, “Are you still breastfeeding her?” Or like, “Why aren’t you breastfeeding her?” So in our culture, it’s important for you to do it.”*

(Participant 2)

Participants also noted that support systems can vary across cultures and that it is important for PPCs to be aware of these support structures when supporting families.

*“So such as maybe I from China so maybe my family will told me what should I do, what should you do, blah, blah, blah like that. So because yeah, they are my family so they maybe influence me a lot so when I ask a parent coach and they told me a different thing, then I will confuse. Oh what should I listen to? I mean, what suggestion should I follow”?*

(Participant 17)

A few respondents noted that, in ideal situations, a peer support intervention, would have concordance between the PPC and the family.

*“Yeah, diversity. I think it’s as important as it is and as important as it should not be either, having someone that represents them that looks like them so having a diverse group of people ‘cause you know I’m Black and it’s not important but it is important to have someone look like me, another Black person. And it’s kind of crazy to say in this day and age that representation is lacking. But it does matter but it shouldn’t see, like if there was a Spanish-speaking family and I try to help them out and it’s like even though they speak English, they probably feel more comfortable, more relaxed if they could have another Spanish-speaking person”*

(Participant 11)

## 4. Discussion

Multiple insights emerged about how a PPC could support healthy infant nutrition and growth. Potential pathways included: providing emotional support, offering education on infant satiety cues, feeding practices, and nutrition, and assisting families in navigating barriers to accessing nutritious foods, particularly among those experiencing FI.

The birth of a child is an exciting and transformative time in a parent’s life; however, parents may face multiple stressors and barriers to supporting their infant’s growth and development. Isolation and mental health challenges are examples of stressors and barriers faced by families with newborns [33,34]. Supporting the emotional wellbeing of parents is critical for promoting optimal growth and development of children. Unfortunately, the mental health of mothers in the U.S. has declined between 2016 and 2023, with elevated rates of poor mental health observed among single parents, mothers with lower educational attainment, and mothers whose child is publicly insured or uninsured [33]. Poor maternal mental health is associated with non-responsive feeding behaviors that can increase a child’s risk of obesity, and chronic maternal depression is positively associated with an increased risk of children becoming overweight [10,11]. A PPC could play an important role in supporting families by providing emotional support through active listening, regular check-ins, or words of encouragement. Our study findings suggest that PPCs who have recently cared for a newborn are particularly well positioned to provide emotional support to families with newborns. Notably, one participant emphasized the importance of considering how to provide emotional support to fathers, who may also face challenges in adjusting to life with a newborn.

Our study also suggests that a PPC could support families with newborns and young infants by providing education and guidance on infant feeding practices. Families commonly expressed concerns about ensuring adequate nutrition, recognizing infant satiety, and introducing healthy foods to their child. Addressing these concerns through trusted, peer-based support may positively influence the growth of children during the critical early months of an infant’s life. The literature indicates that growth during the first 9 months of life can significant affect a child’s risk for obesity, and suboptimal nutrition behaviors may begin as early as 2 months of age [17,18]. Supporting families in identifying satiety cues may be particularly important in promoting healthy weight gain during infancy.

Parental feeding behaviors play a critical role in shaping a child’s weight trajectory, and FI can contribute to parental feeding practices that elevate a child’s risk of obesity [9,12,13]. Although these parental feeding behaviors are modifiable through targeted interventions [35,36], there is a need for approaches specifically tailored for families with limited socioeconomic resources or those that experiencing FI. As suggested by findings from our study, it is critical for interventions to consider the household and community food environments, including the availability and accessibility of local food resources. A PPC serving as an ongoing, trusted source of information on infant feeding and nutrition may help improve the nutrition of infants and support parents in recognizing satiety cues, key factors in promoting healthy growth.

Connecting families experiencing FI or other barriers to accessing nutritious foods is a critical step in improving the nutrition and health of children [37], However, identifying families in need and facilitating appropriate resource connections can be complex. While previous studies have demonstrated that screening for FI is feasible and acceptable among families in pediatric primary care settings, concerns remain regarding stigma, potential consequences of disclosure including referral to child protective services, and ensuring that families are connected to resources [38,39,40,41]. Participants in our study indicated that a PPC could help promote food security by providing information about available resources and addressing concerns about accessing them. Given that FI can be dynamic and may fluctuate over time for families [16,39], PPCs are well positioned to offer longitudinal support and respond to evolving resource needs as identified by families. A common concern voiced by parents was the lack of follow-up after disclosing FI [39,41]. A PPC could help address this gap by providing consistent support and ensuring that families who report food-related needs are connected to appropriate services.

Cultural awareness is essential to consider in the development of the peer parent intervention aimed at supporting the growth and nutrition of infants. In the United States, the prevalence of obesity varies by based on demographic, household, and community factors. As such, obesity prevention strategies should be tailored to the unique strengths, needs, and resources of the communities they serve [8]. Our study suggests that an awareness of a family’s culture, perspectives on infant feeding, and family structure are important elements for a PPC to recognize. These findings are consistent with a scoping review that explored overweight and obesity interventions for children from predominately Hispanic or Black communities. The review found that tailoring interventions to address language barriers, food choices, and family support structures is important when adapting interventions to communities with heterogenous cultures [42]. In our study, participants noted that concordance between peers and families could be beneficial. Similar findings have been observed in other areas of pediatrics, including newborn outcomes [43]; however, concordance may not always be feasible. Incorporating training in cultural humility and the appropriate use of interpreters has been shown to improve communication between physicians and their patients and should be included in the training of PPCs [44,45].

## 5. Limitations

This study has several limitations. First, the data from our study came from a single study site and was limited to participants who spoke English or Spanish and a single clinical site, which may limit the generalizability of findings. Given the sensitive topics explored in the study, such as food insecurity and infant feeding, our results might be influenced by social desirability and recall bias. Only one participant in our sample identified as a father, limiting the ability to fully explore the potential effects of a peer parent intervention among fathers with young infants. Third, our study focused on children ≤6 months of age; thus, findings may not be applicable to families with children at later stages of childhood. There is a chance for selection bias because participation in our study was voluntary. Differences in experiences may exist between families who agreed to participate in the study and families who declined to participate in the study. Finally, while our study reached thematic saturation there is a possibility that not all potential themes were not captured in our study, which may limit the comprehensiveness of our findings.

## 6. Conclusions

A peer parent support intervention might be beneficial in supporting the healthy growth and nutrition of infants by providing (1) emotional support, (2) education on infant feeding and growth, and (3) knowledge about federal and community resources to address FI. Future work should evaluate the feasibility and acceptability of a peer parent coach intervention (Table 6).

## Figures and Tables

**Table 1:** Examples of Semi-Structured Interview Questions.

Main Questions	Probes
To begin, can you tell me a little about yourself and your family?	Where do you live? What do you consider home?
We will be working with peer support coaches as someone who will provide support and education to support healthy infant weight gain. What are some ways a peer parent coach could support the healthy growth and nutrition of families with infants?	How might a peer support coach reinforce/review hunger cues? How might a peer support coach provide support for bottle/breastfeeding? How might a peer support coach provide support provide emotional support to parents?
Were times when you didn’t have enough food, what strategies did your family use to get through these challenging times?	How might a peer support coach reinforce/review hunger cues? What training and resources do you think a peer parent coach would need to support families who experience food insecurity or need help enrolling in WIC or SNAP? What experiences (positive or negative) have you had with WIC/SNAP?

**Table 2:** Sample Demographics.

Characteristic	N (Percentage)
Household food insecurity	11%
Relationship to child	
Mother	17 (94%)
Father	1 (6%)
Parent age (average)	31.1 years
Hispanic	6 (33%)
Race	
Black	6 (33%)
White	5 (28%)
Asian	2 (11%)
Other/Prefer not to respond	5 (28%)
Preferred Language	
Spanish	4 (22%)
English	14 (78%)
Insurance status	
Public	12 (67%)
Private	2 (11%)
Uninsured	4 (22%)
Income	
<$10 k	4 (22%)
$10–20 k	5 (28%)
>$20–40 k	4 (22%)
>$40 k	5 (28%)
Educational level	
<High school	2 (11%)
High school	8 (44%)
Some college	1 (6%)
College	2 (11%)
Graduate	5 (28%)
Enrolled in WIC-yes	10 (56%)
Number of children in home	
1 child	8 (44%)
2 children	4 (22%)
>2 children	6 (33%)

**Table 3:** Theme A: Opportunities for PPC to Provide Emotional Support to Families.

Subtheme	Quote
Subtheme A1: a PPC can provide emotional support through proactive check-in with families	Just to try to just talk or check in up on someone. You can ask if they’re okay, if they need any resources or if they think they’re good. And, you know, if there’s not, or they can just give suggestions on how to make feeding better (Participant 3)
Subtheme A2: Participants described emotional support being most useful when delivered by people in similar stages of life	Or maybe just beyond your stage like with a slightly older child that’s like just gone through what you’re going through. Unless it was like you said that there was a guide that they presented to you, but just hearing that personal experience of other people too. It’s fun and it’s encouraging and it allows you to feel like you’ve got a friend who’s going through it with you. (Participant 1)
Subtheme A3: Empathizing and connecting with participants to provide emotional support	I feel like someone who’s interested in peer support should be patient, understanding, encouraging, easy to talk to. (Participant 12)

**Table 4:** Theme B: Educational Support Focused on Infant Nutrition.

Subtheme	Quote
Subtheme B1: Participants described a preference for infant nutritional education (feeding patterns and preparation of formula)	Some parents start earlier, some parents start later just to have that more like okay, like every child is different instead of trying to follow a rulebook. You have to—I wish I had someone to tell me that, no, you have to watch your child for cues if they’re ready instead of like okay, this is at six months or this is a year, they should be at this point at this time and that’s not true for every child. (Participant 11) Like information about cluster feeding would help. Right now I feed him for thirty minutes, fifteen minutes on each side (Participant 12) Because there’s a lot of basics even though I read a lot of books before she was born. There’s a lot of stuff that I just didn’t know and I think having someone to go to for those kinds of things would’ve been helpful. (Participant 13)

**Table 5:** Theme C: PPC can provide knowledge and facilitate access of resources.

Subtheme	Quote
Subtheme C1: PPC can address to participation in federal nutrition programs	Mmm, pues sí, pues a veces no estamos informados. A veces pensamos que es difícil, uh, eso del programa del WIC, pero pues a veces el instructor pues si tienes, uh, si puede ayudar, pues, sí es mejor porque, pues, te dan una fórmula o… A veces tienes como preguntas y pues pienso que ellos te las pueden sacar. Como te digo, a veces uno piensa que todo es difícil así, pero pues ya luego te dicen: “No, haz esto así, y en esto llena esta hoja, nada más tienes que entregar esto” y pues es más fácil. Son las cosas… a veces uno piensa que son difíciles pero no. (Participant 14) ○ English translation: “Mmm, well yes, sometimes we’re not informed. Sometimes we think it’s diffiuclt, u,n that WIC program, but well sometimes the instructor, well if you have one, uh, if they can help, well, it is better becaue, well, they give you a formula or … Sometimes you have questions and well I think they can anaswer them. Like I said, sometimes you think everything is dificult like that, but then they will tell you: ‘No, do this like this, well just fill out this form, you only have to turn this in,’ and well, it’s easier. These things… sometimos you think they’re difficult, but they’re not.”
Subtheme C2: Clear information about participating in programs	Pues más que nada informarlos bien, como, darles exactamente la información correcta. Porque hay veces que te meten miedo, como que: “Ay, si agarras esto, eh, estos programas, este, pues a futuro te traen consecuencias o problemas.” Hay gente que así-así le hace. Entonces más que nada informar a la gente con la verdad. Como, los procesos, qué se necesita, el tiempo que necesitas para los trámites y-y todo eso. Más que nada hablar con la verdad. Con la verdad y-y ser lo más, lo-lo más explícitos porque a veces, este, uno no entiende, como, toda la información o a veces te la hacen como muy revoltosa. Entonces entre menos palabras y más claros, mejor. (Participant 15) ○ *English translation: “Well, more than anything, to inform them well, like, give them exactly the correct information. Because sometimes they scare you, like: ‘Oh, if you receive this, uh, these programs, uh, well in the future it’ll bring you consequences or problems.’ There are people who do do that. So more than anything, inform people with the truth. Like, the process, what’s needed, the time you need for the paperwork and—and all that. More than anything, speak the truth. With the truth and—and be as—as explicit as possible because sometimes, uh, you don’t understand, like, all the information or sometimes they make it all really confusing. So the fewer words and the clearer, the better.”*
Subtheme C3: Awareness of resources in case needs arise	Definitely you know having an awareness like again ‘cause it might not be something that they, people they are helping even know that they need help with. Like “Oh yeah, we’ve always struggled having food in the house” or something like that, just having that awareness or we have these things in place to help in the event that it happens. (Participant 9)

**Table 6:** The importance of understanding a family’s culture and lived experience.

Subtheme	Quote
Subtheme D1: a PPC should be aware of and open to people from different backgrounds and cultures	Ajá. Pues, como le digo, más que nada preguntar, preguntar, cómo-cómo se vive, este, qué es lo que comen. Porque en sí, como uno de mexicano come ciertas cosas que otras personas de otros lugares no comen, entonces es, como, variable la alimentación de-de cada familia por eso mismo, por el lugar donde vienes. Como, desde los que nacieron aquí, los que viven aquí de, de pues demuchos años atrás. Entonces yo pienso que se deberían de enfocar más, como, en lo que consume cada familia. (Participant 15) English translation: “Well, as I was saying, it’s mostly about asking questions, asking how they live, what they eat. Because, as a Mexican, I eat certain things that people from other places don’t eat, so the diet of each family varies depending on where they come from. Like, those who were born here, those who have lived here for many years. So I think they should focus more on what each family consumes.”
Subtheme D2: Expectations around infant feeding and nutrition is driven by cultural norms	I don’t want to tell you every culture or whatever, but ... and black people, we tend to feed our children at a younger age things they definitely do not need (Participant 18)
Subtheme D3: Important to understand the different family structures	.... it’s different. Because back in Kenya, we have like great support systems. You have your mother, your husband, you find out that like as a mother, you give back. You just take care of the baby for a moment. So, that keeps you healing and in recovery, so that way you don’t have to stress about anything. And here, like, it’s different here. It’s just like, you have some friends, and everyone’s like, busy here, So, you find out sometimes it can be a little bit. So, it has ups and downs here. So, you bring like, the mother would be in a position to be like, really really close, but like, you don’t have to worry about anything, you know? (Participant 16)

## Data Availability

The datasets generated and analyzed during the current study are not publicly available due to the sensitive and identifiable nature of qualitative interview data, but de-identified data are available from the corresponding author on reasonable request.
